# Enhanced Interfacial and Mechanical Properties of PBX Composites via Surface Modification on Energetic Crystals

**DOI:** 10.3390/polym11081308

**Published:** 2019-08-05

**Authors:** Chengcheng Zeng, Zhijian Yang, Jianhu Zhang, Yubin Li, Congmei Lin, Guansong He, Xu Zhao, Shijun Liu, Feiyan Gong

**Affiliations:** Institute of Chemical Materials, CAEP, Mianyang 621900, China

**Keywords:** grafting, energetic crystals, PDA, mechanical properties

## Abstract

The mechanical properties of composites are highly dependent on the interfacial interaction. In the present work, inspired by marine mussel, the adhesion between energetic crystals of 1,3,5-triamino-2,4,6-trinitrobenzene (TATB) and polymer binders was improved. Three types of linear polymeric agents of glycidyl azide polymer (GAP), polyethylene glycol (PEG), and polytetramethylene ether glycol (PTMEG) were grafted onto TATB particles bridged through polydopamine (PDA) films. SEM images showed that 5% grafting contents could evidently form roughness shells on the surface. With a reinforcement at the interface produced by grafting shells, the mechanical properties of polymer-bonded explosives (PBXs) exhibited outstanding mechanical performance, especially for the PTMEG-grafting sample. Examined by the contact-angle test, the PTMEG-grafting sample possessed a value of polar component similar to that of fluoropolymer, leading to an excellent wettability of the two phases. Additionally, different contents of PTMEG were grafted to reveal that the mechanical properties could be improved even with content as little as 0.5 wt.% PTMEG. These results might highlight a correlation between interfacial interaction and macroscopic properties for mechanically energetic composites, while providing a versatile route of grafting on highly loaded composites.

## 1. Introduction

Polymer-bonded explosives (PBXs), including energetic materials (EMs) and polymer binders, attracted extensive attention due to their high chemical energy and insensitivity in both military and civil fields [1,2,3]. In this multi-phase system, the interfaces between energetic crystals and polymer substrates, including solid–solid interfaces and solid–gas interfaces produced by small holes, could be critical for practical use. The weak interface interaction seriously affects further surface coating and the interfacial strength of the components, resulting in the damage of PBXs in the face of complex mechanics and temperature shock, which restricts their safety and reliability [4,5].

In the multi-component PBX composite materials, interfacial debonding caused by the large property differences and the incomplete contact between explosive particles and binders leads to the poor mechanical performance of PBXs [6,7,8,9]. Usually, the interfacial work can be effectively improved by introducing a coupling agent on the explosive’s surface, which acts as a “bridge” molecule [10,11,12]. Zhang et al. [10] revealed a new coupling mechanism of silane coupling agents between binders and 1,3,5-triamino-2,4,6-trinitrobenzene (TATB) by using dissipative particle dynamics simulations. The bonding agent could preferentially attach TATB’s affinity structural units and adhesive at the interface, while the proportion of non-affinity units decreased. Coupling agents can not only produce hydrogen bonds with amino or nitro groups on the surface of explosives, but also interact with fluoropolymer molecules, thus improving the interfacial strength to a certain extent. However, limitations of simply introducing coupling agents to improve the interfacial properties may be caused by incomplete coating and sharp selectivity on explosives.

In the past decade, deep insights were widely gathered into dopamine for surface modification. Dopamine can attach to the surface of most inorganic and organic materials due to its robust molecular adhesion through catechol–substrate interactions [13,14,15]. Dopamine forms a nano-thick polydopamine (PDA) layer on the surface of the materials through its own oxidative self-polymerization reaction, by forming covalent bonds, π–π conjugation, and other forces [16]. Polymerization of dopamine can be carried out in aqueous solution with a mild reaction, which is suitable for modifying the surface of explosive crystals. Gong et al. [17] found that a PDA layer wrapped on 1,3,5,7-tetranitro-1,3,5,7-tetraazacyclooctane (HMX) could form an effective coating and postpone the high-temperature transformation of HMX. A further study reported that the thickness of PDA shells could be adjusted by changing the polymerization time of dopamine, and mechanical properties of TATB-based PBXs were proportional to the thickness [18]. Moreover, the structure of PDA contains a number of hydroxyl, amino, and other reactive groups, which can be used as secondary reaction platforms for the grafting reaction. The interface properties between the explosive and adhesives can be improved by selecting specific polymers as grafting objects, further enhancing the mechanical properties of PBXs. Compared with physical coating, chemical grafting is less used for polymers, which is beneficial for maintaining the energetic level of the energetic system, and the grafting layer is more reliable and compact.

Based on the abovementioned issues, PDA layers with active groups were pre-modified on TATB, and three types of linear polymers with different molar mass were grafted by using –NCO groups as bridging intermediates. As a result of surface modification of TATB, the interface between energetic particles and binders was reinforced to improve the mechanical performance (including dynamic and static tests) of energetic composites. A joint achievement of strengthening and toughening upon introducing the polymeric shells would effectively promote the practical use of PBX composites. The proposed grafting method may provide new insights into the interface reinforcement in multi-component composites.

## 2. Materials and Methods

### 2.1. Materials

TATB (purity 98%) was synthesized by the Institute of Chemical Materials, CAEP, Mianyang, China and used without further purification. Dopamine hydrochloride (purity 98%) and (hydroxymethyl) aminomethane (Tris, purity 99%) were purchased from Sigma-Aldrich (St. Louis, MI, USA). Ditin butyl dilaurate (DBTDL, purity 95%), used as a catalyst, and toluene-2,4-diisocyanate (TDI, AR), acting as an intermediate in the grafting reaction, were purchased from Aladdin Chemical Ltd. (Shanghai, China). Interface-reinforced polymers, including polytetramethylene ether glycol (PTMEG, number average molecular weight (Mn) = 1000 g·mol^−1^), polyethylene glycol (PEG, Mn = 600 g·mol^−1^), and glycidyl azide polymer (GAP, Mn = 3600 g·mol^−1^) were also bought from Aladdin Chemical Ltd. (Shanghai, China). Other chemicals reagents were of commercial analytical purity (AR) and used as solvents. The fluoropolymer binder (F_2314_) used as the polymer binder in this work was a copolymer of chlorotri-uoroethylene (CTFE) and vinylidene fluoride (VDF), and provided by Zhonghao Chenguang Chemical Industry Co. Ltd., Zigong, China.

### 2.2. Coating of TATB with PDA and Interface-Reinforced Polymers

**Figure 1:**
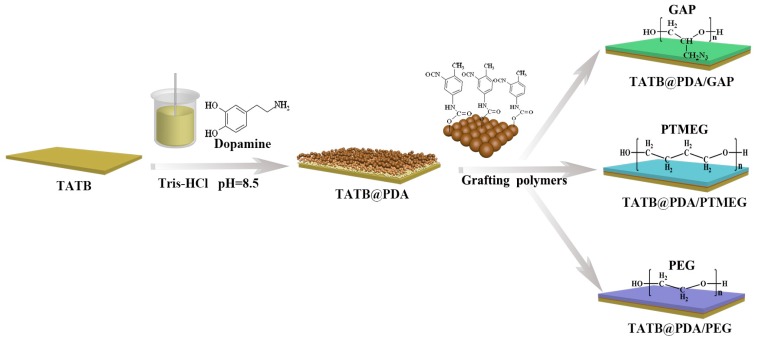
Schematic diagram of preparation process for surface grafting powders.

The schematic diagram of grafted TATB is displayed in Figure 1, which facilitates a better understanding of the preparation process. As illustrated in a previous article [17], 10 mM Tris was firstly dissolved into deionized water, adjusting the solution to a pH of 8.5 by introducing HCl. Then, TATB crystals and 2.0 g·L^−1^ dopamine were added to the fresh weak alkaline solution, stirring at 600 rpm. After 6 h for PDA coating, the color of TATB@PDA (TP) changed into dark brown, which is different from that of pristine TATB, suggesting an achievement of the self-polymerization of dopamine.

Then, three linear polymers were coated by the reaction between TDI (acting as intermediary molecules) and PDA shells (containing –OH and –NH_2_ groups). Two –NCO groups at the interval site of TDI would react separately with the active groups of PDA and those of the three selective polymers under specific ambience. The detailed preparation of double core–shell TP@GAP (TPG), TP@PTMEG (TPP), and TP@PEG (TPE) was as follows: TP powder was treated in a vacuum oven at 80 °C for 0.5 h before use, and then 100 g of TP was put into a double-layered vessel with 300 mL of butyl acetate. After 30 min of stirring, DBTDL and TDI were added at a ratio of 1.5:1. After a 1-h reaction, the TDI molecule was successfully grafted onto the TATB surface through PDA layers, as explained by the second arrow in Figure 1. Next, the suspension solution was washed and filtered several times to remove dissociative TDI. The powders were transferred into a vessel containing butyl acetate. Under vigorous stirring, the same amount of DBTDL was added again, and then 5 g of linear polymer (GAP, PTMEG, and PEG) dissolved in butyl acetate was introduced into the suspension solution. After a 1-h reaction, the second reaction of polymer grafting was accomplished. Three types of core–shell configuration, as expressed in Figure 1, were obtained by washing and filtering several times and drying in vacuum for 6 h. Since the added TDI was excessive, the grafted polymer content was 5 wt.% (calculated based on 100 g of TATB).

### 2.3. Preparation of TATB-Based PBX

A non-aqueous solvent granulation method was used to prepare PBX composites composed of modified TATB and fluoropolymer (F_2314_), and then the molding powders were dried at 60 °C for 24 h to eliminate solvent. TATB-based PBXs generated by grafting GAP, PTMEG, and PEG were labeled as TPGF, TPPF, and TPEF, respectively. PBX consisting of neat TATB was labeled as TF. A list of the abbreviations used is provided in Table 1. After all preparation was done, the molding powders were pressed into different pellets for the different tests.

### 2.4. Characterization Method

The morphologies of initial TATB and grafted TATB were observed by using scanning electron microscopy with a scanning signal of SE2 mode (SEM, ΣIGMA-HD-0129, Zeiss, Oberkochen, Germany). Because energetic crystals are easily destroyed by long-term electronic beam scanning, an acceleration voltage of 3 kV and a fast scanning speed had to be taken into account. Fourier-transform infrared spectroscopy (FTIR, Thermo, Nicolet 6700, Waltham, MA, USA) was conducted using DTGS KBr with a wavenumber range of 400–4000 cm^−1^. Raman spectra were collected to analyze the surface chemical structure by using a LabRAM HR Evolution system (HORIBA Jobin Yvon, Paris, France) with an He–Ne ion laser (514.0 nm) as the source. The thermodynamic properties of samples were characterized by differential scanning calorimetry (DSC, TGA/DSC 2, METTLER) at a heat rate of 10 °C·min^−1^ under nitrogen atmosphere (from 50 °C to 500 °C). The static mechanical tests, including the Brazil test and compressive test, were performed with a universal testing machine (INSTRON 5582, Norwood, MA, USA), by using the pellets with dimensions of Φ20 mm × 6 mm and Φ20 mm × 20 mm, respectively. Dynamic mechanical analysis (DMA) using a three-point bending mode was conducted by an apparatus (DMA 242C, Netzsch, Selb, Germany), with a size of 30 mm × 10 mm × 2 mm at different temperature (60 and 75 °C) and different applied stress (6, 7, and 8 MPa). Each mechanical test was performed three times.

The contact angle of modified TATB with water and diiodomethane droplets was measured by an optical contact-angle measuring instrument (DSA-20, Cruss, Hamburg, Germany). For the accuracy of the test, each droplet was measured two times to obtain average values with calculated standard deviations. Surface energy (*γ*), which consisted of polar components (*γ^p^*) and dispersion components (*γ^d^*), was described by Equations (1) and (2).

(1) $$\gamma_{\mathrm{ls}}=\gamma_{s}-\;\gamma_{l}\cos\theta ,$$

(2) $$\gamma_{\mathrm{ls}}=\gamma_{l}+\gamma_{s}-2\sqrt{\left(\gamma_{s}^{p}\gamma_{l}^{p}-\gamma_{s}^{d}\gamma_{l}^{d}\right)},$$

where subscripts *S* and *L* of the parameters represent solid and liquid, respectively. The relationship between surface energy (*γ*) and contact angle (*θ*) is expressed in Equation (3), which was extracted by Owen [19].

(3) $$\gamma_{l}\left(1\;+\cos\theta \right)=2\left(\sqrt{\gamma_{s}^{p}\gamma_{l}^{p}}+\sqrt{\gamma_{s}^{d}\gamma_{l}^{d}}\right).$$

## 3. Results and Discussion

### 3.1. Morphology and Chemical Structure Characterization of Polymer-Grafted TATB

**Figure 2:**
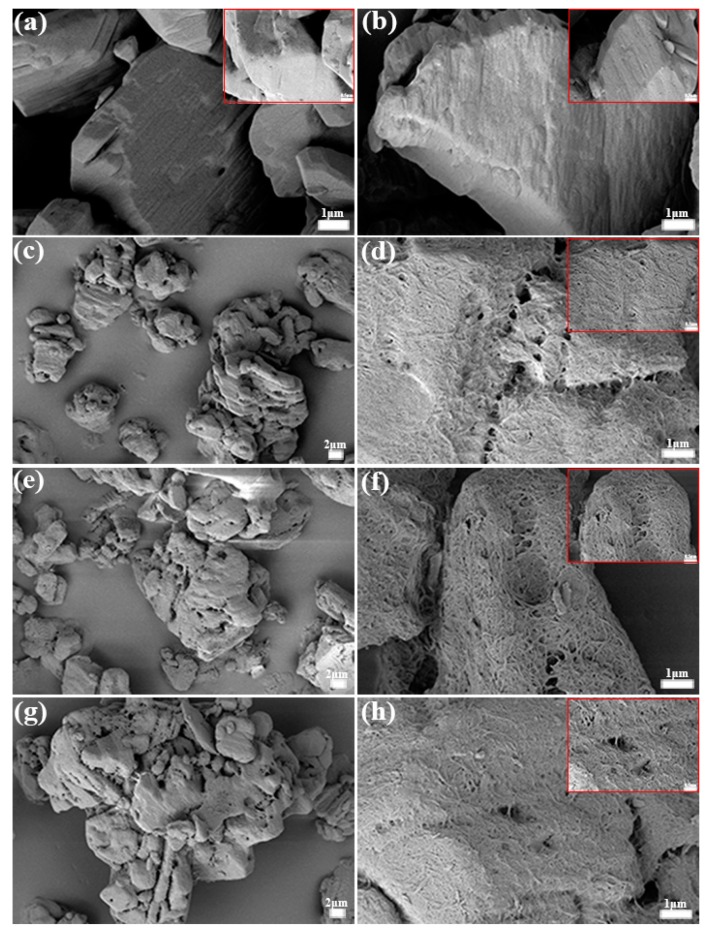
SEM images for (**a**) pristine 1,3,5-triamino-2,4,6-trinitrobenzene (TATB), (**b**) polydopamine (PDA)-coated TATB, and PDA-coated TATB with (**c**) glycidyl azide polymer (TPG), (**e**) polytetramethylene ether glycol (TPP), and (**g**) polyethylene (TPE). (**d**,**f**,**h**) (including the corresponding inserts) high-magnification images for the surfaces of modified TATB shown in (**c**,**e**,**g**), respectively.

The morphologies of initial TATB, TATB@PDA, and TP-grafting powders were examined by SEM measurements, as shown in Figure 2a–h. The surface of the TATB, with a diameter of about 25 μm, displayed some ravines and small holes, which could be caused by different crystallization processes. As can be seen from Figure 2a,b, the surface of PDA-coated TATB exhibited a rougher surface than that of TATB, which was mainly related to the deposition of PDA onto the surface of crystals via particle aggregation [20]. Figure 2c,e,g, as well as their respective inserts, show the surface morphologies of TPG, TPP, and TPE. Noticeably, the linear polymers grafted onto TP powders, and the molecular chains interweaved to form nest-like shells. In addition, it was difficult to distinguish the three types of grafted samples only from SEM pictures and the high-magnification inserts, even though differences existed in the molecular structures and molecular weights of the grafting polymers.

**Figure 3:**
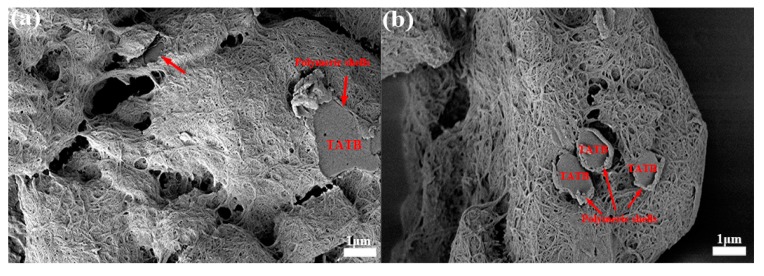
SEM images for polymeric shells with glycidyl azide polymer (GAP) grafting (**a**) and polytetramethylene ether glycol (PTMEG) (**b**).

To confirm the core–shell configuration, SEM images of TATB crystals with obvious polymeric shells are depicted in Figure 3a,b. As indicated by the red arrows, the polymeric molecules cross-linked with each other to constitute compact shells on the TATB surface, determining that the grafted powders were core–shell structures. Since the cross-section of the films was exposed, the thickness of the polymeric coating was approximately 50 nm.

**Figure 4:**
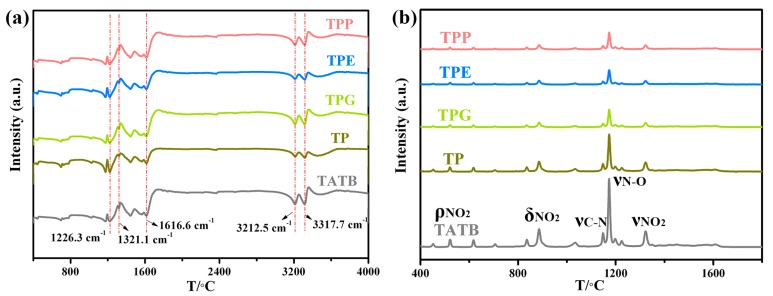
Fourier-transform infrared (FTIR) (**a**) and Raman (**b**) spectra of TATB with and without surface modification.

The chemical structure analysis on the surface of initial TATB and the modified powders is displayed in Figure 4. As can be seen from Figure 4a, FTIR measurements were insensitive when it came to distinguishing the chemical groups due to the complex background of TATB. The absorbing peaks at 3317.7 and 3212.5 cm^−1^ were attributed to the stretching vibration of –NH_2_, and the peak at 1616.6 cm^−1^ was the in-phase vibration of C=C. Moreover, the –NO_2_ stretching vibration and C–N stretching vibration were identified at the peaks of 1321.1 cm^−1^ and 1226.3 cm^−1^. The Raman investigation is shown in Figure 4b, from which the obtained Raman spectra, mainly including nitro groups, were consistent with previous literature, owing to ρ_NO2_, δ_NO2_, ν_C-N_, ν_N-O_, and ν_NO2_ [21]. After being coated with PDA, the intensity of the characteristic peaks for TP powder became weak, while the intensity of the main peaks for modified powders clearly decreased upon further grafting with linear polymers. Therefore, the surface modification did not change the chemical groups of TATB.

### 3.2. Mechanical Properties of Polymer-Grafted TATB

To reveal the divergence of mechanical properties caused by the different kinds of linear polymers, the static mechanical test and three-point bending test were used, which could easily and effectively reflect the mechanical properties in different TATB-based composites.

**Figure 5:**
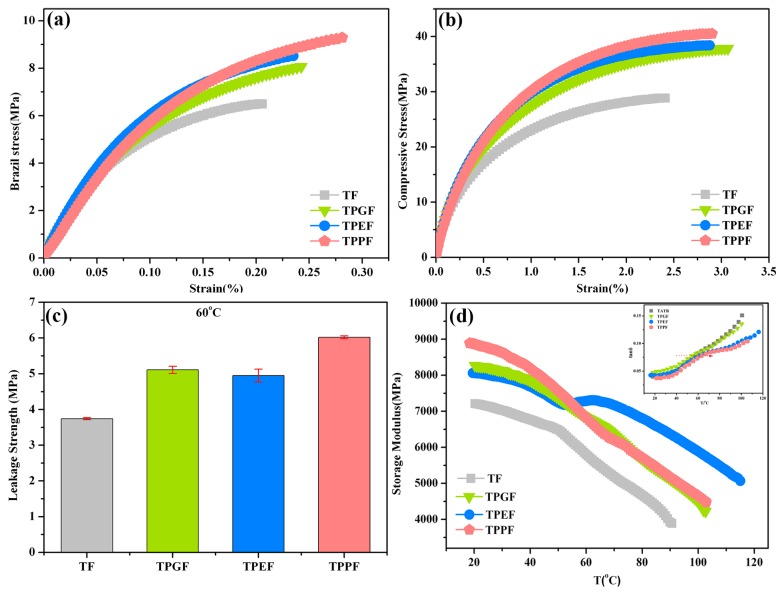
Stress–strain curves of (**a**) Brazil test, (**b**) compression test, (**c**) leakage strength, and (**d**) storage modulus for TATB-based polymer-bonded explosive (PBX) with and without grafting; the insert of (**d**) shows the glass transition.

Typical stress–strain curves of the Brazil test and compressive test at room temperature are presented in Figure 5a,b. The Brazil test, a typical static mechanical test, is a simplified tensile test, which may be the most effective method to evaluate brittle fracture stress by using disc-like samples. The maximum stress and elongation of all grafted samples (reinforced by 5 wt.% GAP, PTMEG, and PEG) were remarkably increased at room temperature, due to the roughness increase following the polymeric grafting. The coarse shells may enable increased interaction with the fluoropolymer, thereby efficiently improving the interfacial stress transfer [22]. Compared with the TATB/F_2314_ composite, the maximum Brazil strength of TPPF was increased from 6.64 ± 0.18 MPa to 9.3 ± 0.05 MPa (by 40.9%), and the compressive strength was increased from 28.91 ± 0.25 MPa to 40.53 ± 0.42 MPa (by 40.1%). Surprisingly, the fracture elongation was significantly improved at the same time.

The leakage strength was similar to the maximum Brazil strength, except that it experienced different temperatures. Figure 5c shows the leakage strengths of all sample with and without grafting at 60 °C. The values of all grafted samples evidently increased, especially for the TPPF composite with an increment of 61%, compared with the TF sample. This is reasonable because the strong interfacial adhesion of the polymer coating is believed to inhabit the slip of F_2314_, resulting in a better mechanical performance [23].

DMA testing is a valid method to examine the effect of polymeric shells on the interface, which is of great significance for the design and application of composite materials [24,25]. The storage modulus (E’) of all samples was determined from the three-point bending mode, which can better reflect the resistance to deformation, and the curves of E’ vs. T are shown in Figure 5d. During the investigated range (20–120 °C), the E’ of the TF sample firstly decreased slowly, and then started reducing quickly at about 60 °C, resulting from the glass transition of F_2314_ binders. It was noteworthy that the 5% grafting of polymers onto TATB particles remarkably increased the E’ across the whole temperature range, indicating a strong interfacial interaction between the TATB crystal and fluoropolymer binder [16]. As evidenced in the insert of Figure 5d, the curves of tan δ vs. T reflected the glass transition of F_2314_, from which the unintelligible peak was due to the low content of fluoropolymer. Identified by the red arrow, every modified sample showed a smaller delay of glass transition compared to that of TATB with a glass transition at 58 °C. The existence of the polymeric shells significantly increased the overall cross-link density, which restricted the mobility of F_2314_ molecules [26]. Overall, an outstanding E’ of about 9000 MPa in the PTMEG-grafted TATB indicated the dramatic reinforcement in the composites.

### 3.3. Surface and Thermodynamic Properties of Polymer-Grafted TATB

**Figure 6:**
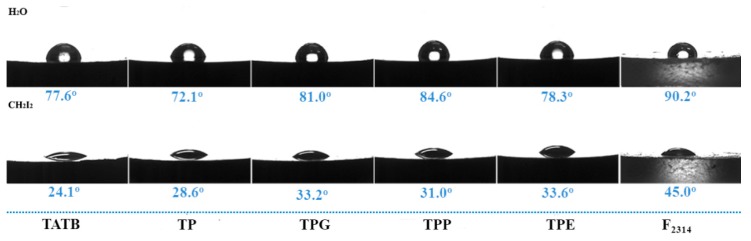
Contact angles using water and diiodomethane for different grafted TATB samples.

Based on the above results, it was found that the linear polymers with different molecular weights could not be distinguished from the SEM pictures, but their strengths and moduli were quite different. In order to further understand the relationship between the material interfaces and their mechanical properties, the static contact angle test was used to calculate the surface energy. As shown in Figure 6, the water and diiodomethane contact angles of TATB were 77.4° and 24.1°, respectively, showing the low surface energy of TATB [26]. After PDA coating, the angles changed to exhibit better water wettability, due to the functional groups on the surface of PDA [27]. When the three polymers were grafted, both contact angles increased, demonstrating that the surface modification of TATB would significantly change the surface properties. Furthermore, the F_2314_ pellets were also treated as references to assist the measurement. All of the contact angles and their corresponding standard deviations are depicted in Table 2, from which it can be seen that there was little variation in the values for the same sample, illustrating the accuracy of the measurement.

Water and diiodomethane, with a large difference in surface energy, and its components were used to count the components. The *γ*_l_^p^ and *γ*_l_^d^ of water are 51 and 21.8 mN·m^−1^, and those of diiodomethane are 0 and 50.8 mN/m [28]. According to Equations (1)–(3), the calculated surface energy and its components for grafted TATB are listed in Table 2. The surface energy increased from 49.49 mN·m^−2^ for TATB to 50.05 mN·m^−2^ for TP after PDA functionalization, which is attributable to the π–π stacks and hydrogen bonding during dopamine polymerization [29]. Comparing the grafted TATB samples, the surface energy decreased upon decreasing the polar and dispersion components. Additionally, the contact angle of F_2314_ was also measured to figure out the relationship between the interfacial interaction and mechanical properties. As can be seen from Table 1, only the polar components of the TPP and F_2314_ samples were approximate among all the grafted samples. According to the “like dissolves like” principle, when the polarity of the two phases was approximated, excellent dispersion and wettability between the two phases could be obtained [30,31]. Overall, PTMEG, located on the PDA shell, firmly bonded to TATB and dispersed uniformly within the fluoropolymer, playing an interlocking role at the interface. Therefore, the TPPF sample owed its impressive mechanical properties to the strong interfacial adhesion with the matrix, resulting in a high load transfer efficiency [28].

**Figure 7:**
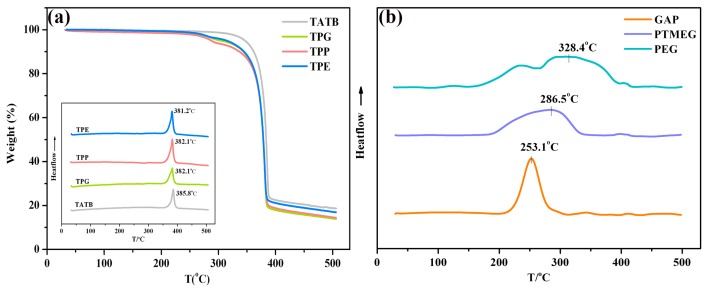
(**a**) Thermogravimetric analysis (TGA) curves of TATB with and without grafting; the inset shows the differential scanning calorimetry (DSC) curves; (**b**) DSC curves of GAP, PTMEG, and PEG polymers.

The TGA curves and corresponding DSC results of modified TATB are shown in Figure 7a. From Figure 7a, TATB displayed a one-step reaction, starting to decompose at about 310 °C, with a weight loss of 81%. Unlike raw TATB, grafted TATB samples with different types of polymers exhibited a two-stage loss, i.e., the decomposition of polymers and TATB. The DSC curves of the three polymers are displayed in the Figure 7b, from which it can be seen that the three polymers presented different heat-release curves. Owing to the energetic –N_3_ groups, GAP decomposed fiercely to produce a sharp peak with a decomposition temperature of 253.1 °C. For PTMEG and PEG, the whole decomposition process occurred over a wide range, indicating a slow thermal reaction. Overall, this was due to the latent heat provided by the polymers in the early decomposition process, during which the peak temperature of TATB advanced from 385.8 °C to 381.2 °C, as shown in the insert of Figure 7a. Therefore, the grafted polymers of GAP, PTMEG, and PEG may induce the decomposition of TATB when increasing temperature.

### 3.4. Morphology and Mechanical Characterization for PTMEG-Grafted TATB

**Figure 8:**
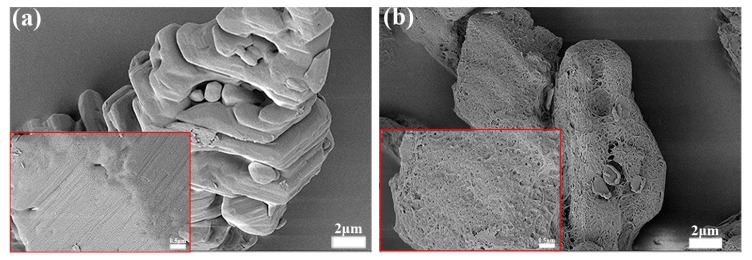
SEM pictures of (**a**) TPPF-0.5 (0.5% PTMEG grafting) and (**b**) TPPF-5 (5% PTMEG grafting) with magnified pictures in the inserts.

Energy levels must also be taken into account for energetic composites; thus, the content of polymers should not be high, usually with a mass fraction of less than 1% [32]. Based on the previous conclusions, a low content of 0.5% for PTMEG was grafted to study the effect of shell thickness on the mechanical properties. The SEM pictures of Figure 8a,b show the surface morphologies for sample TPPF-0.5 (0.5% PTMEG grafting) and sample TPPF-5 (5% PTMEG grafting), respectively. From the magnified pictures inserted in Figure 8, the surface of TPPF-0.5 displayed a thin coating surface, which was almost the same as that of TP powders (shown in Figure 2b), because of the small amount of grafting and the non-perfection of the TATB crystal. On the contrary, the surface of sample TPPF-5 was completely rough with a thicker coating produced by the polymeric shell.

**Figure 9:**
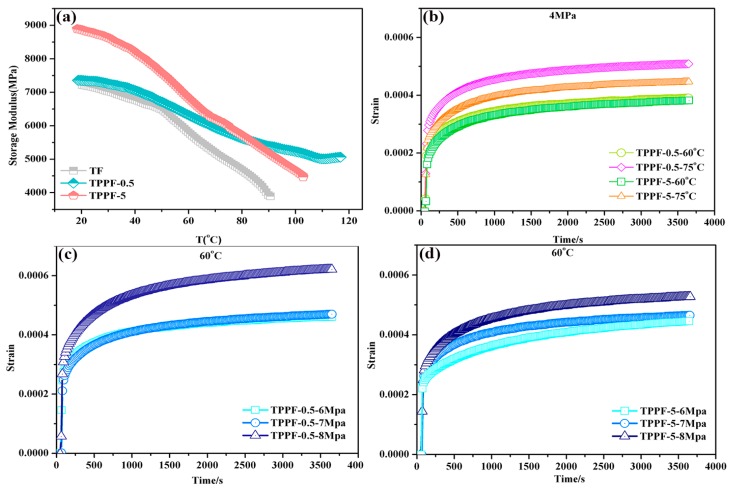
(**a**) Storage modulus, and (**b**–**d**) the creep response under different conditions for TPPF-0.5 and TPPF-5.

Figure 9a shows the dependence of the storage modulus (E’) on the temperature for different thicknesses of grafting PBXs. As can be seen from the figure, the coating thickness was positively correlated with the storage modulus. The E’ of sample TPPF-0.5 was slightly higher than that of sample TF, indicating that PTMEG indeed improved the interfacial interaction. TP crystals with different thickness shells of PTMEG restricted the movement of the fluoropolymer, especially at high temperatures (above 50 °C), resulting in a higher storage modulus compared to that of sample TF [33].

Moreover, the temperature dependence of the creep response for the two samples was investigated. Figure 9b–d show the creep resistances at 60 °C and 75 °C under typical stresses of 6 MPa, 7 MPa, and 8 MPa. As displayed in Figure 9b–d, the strains increased with increasing temperature and stress for both TPPF-0.5 and TPPF-5. For the same sample, the temperature and pressure increases would accelerate the migration of molecular chains, thus leading to an increase in the macro-deformation. As expected, TPPF-5 exhibited better creep resistance compared with TPPF-0.5, probably due to the increasing roughness induced by the grafting layer on the surface of the TATB, as well as the strength of the interfacial interaction with the fluoropolymer [34]. The interweaving molecular chains, which formed the polymeric shells, could give rise to a better resistance of movement, which contributed to low strains.

**Figure 10:**
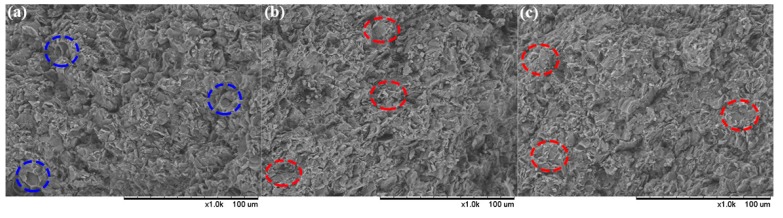
SEM pictures of fracture surfaces for TATB-based PBXs: (**a**) TF, (**b**) TPPF-0.5, and (**c**) TPPF-5.

It is believed that a reinforcement interface was formed between F_2314_ and the TATB matrix so as to gain the exceptional mechanical behaviors, which was confirmed by the fracture surface morphologies of PBXs, as shown in Figure 10. Because of the weak interface, the holes, identified by the blue circles in Figure 10a, came from the separation of the TATB from the matrix. It is suggested that the main fracture mechanism of the TF sample was interfacial debonding between TATB and F_2314_. After PTMEG grafting (Figure 10b,c), the main fracture mode was changed to fluoropolymer breakage, indicating an improvement in the interfacial adhesion strength compared to F_2314_ itself, which was consistent with the previous results.

## 4. Conclusions

In summary, strong adhesion produced by the PDA coating layer was firstly used for TATB modification, and three types of polymers (GAP, PEG, and PTMEG) were consequently grafted via a reaction with the active functional groups of PDA. The grafted polymers obviously changed the surface morphology of TATB, as presented by the SEM images. Specifically, the Brazil strength of PTMEG-grafted PBXs increased by 40.9%, and the compressive strength increased by 40.1%, due to the excellent interfacial interaction between the modified TATB and F_2314_. The storage modulus also showed an outstanding value of 9000 MPa at room temperature compared to the other samples. Multiple PTMEG-grafting samples were prepared to prove that the thickness of shells is positively correlated with the mechanical properties. Above all, the grafting method mentioned for the energetic composites might provide new insight into interface designs for other multi-component composites.

## Figures and Tables

**Table 1:** All of the abbreviations for the modified 1,3,5-triamino-2,4,6-trinitrobenzene (TATB) samples. PDA—polydopamine; GAP—glycidyl azide polymer; PEG—polyethylene; PTMEG—polytetramethylene ether glycol; F_2314_—fluoropolymer.

Sample	Abbreviations for Grafting Powders	Sample	Abbreviations for Composites
TATB	TATB	TATB/F_2314_	TF
TATB@PDA	TP	TATB@PDA/F_2314_	TPF
(TATB@PDA)@GAP	TPG	(TATB@PDA)@GAP/F_2314_	TPGF
(TATB@PDA)@PTMEG	TPP	(TATB@PDA)@PTMEG/F_2314_	TPPF
(TATB@PDA)@PEG	TPE	(TATB@PDA)@PEG/F_2314_	TPEF

**Table 2:** Contact angles and surface energies of different droplets on modified TATB and F_2314_ pellets.

Sample	Contact Angle (°)		Standard Deviation		Surface Energy (mN·m^−2^)		
	Water	Diiodomethane	Water	Diiodomethane	*γ*_s_^p^	*γ*_s_^d^	*γ*_s_
TATB	77.63 ± 0.77	24.10 ± 0.50	1.09	0.71	3.02	46.47	49.49
TP	72.05 ± 0.65	28.53 ± 0.58	0.92	0.81	5.25	44.82	50.07
TPG	80.95 ± 0.45	33.20 ± 0.80	0.64	1.13	2.62	42.85	45.47
TPP	84.60 ± 0.35	31.00 ± 0.30	0.49	0.42	1.56	43.80	45.36
TPE	78.30 ± 0.80	33.60 ± 0.40	1.13	0.57	3.46	42.67	46.13
F_2314_	90.20 ± 0.45	45.00 ± 0.62	0.64	0.88	1.21	37.01	38.22
